# Structural basis of the amidase ClbL central to the biosynthesis of the genotoxin colibactin

**DOI:** 10.1107/S2059798323005703

**Published:** 2023-08-10

**Authors:** Prabhanshu Tripathi, Jarrod J. Mousa, Naga Sandhya Guntaka, Steven D. Bruner

**Affiliations:** a Department of Chemistry, University of Florida, Gainesville, FL 32601, USA; McGill University, Canada

**Keywords:** natural product biosynthesis, amidases, colibactin, ClbL, microbiome

## Abstract

Colibactin is a genotoxic natural product produced by select commensal bacteria in the human gut microbiota. The compound is a bis-electrophile that is predicted to form interstrand DNA cross-links in target cells, leading to double-strand DNA breaks. The biosynthesis of colibactin is carried out by a mixed NRPS–PKS assembly line with several noncanonical features. An amidase, ClbL, plays a key role in the pathway, catalyzing the final step in the formation of the pseudodimeric scaffold. ClbL couples α-aminoketone and β-ketothioester intermediates attached to separate carrier domains on the NRPS–PKS assembly. Here, the 1.9 Å resolution structure of ClbL is reported, providing a structural basis for this key step in the colibactin biosynthetic pathway. The structure reveals an open hydrophobic active site surrounded by flexible loops, and comparison with homologous amidases supports its unusual function and predicts macromolecular interactions with pathway carrier-protein substrates. Modeling protein–protein interactions supports a predicted molecular basis for enzyme–carrier domain interactions. Overall, the work provides structural insight into this unique enzyme that is central to the biosynthesis of colibactin.

## Abstract 2

Insight into biosynthetic pathways in the human microbiome can provide details of host–microbe interactions and beneficial symbiosis. In this report, the structure and function of a key bond-forming enzyme in the biosynthesis of the human genotoxin colibactin is presented.

## 1. Introduction

The human body hosts a complex community of microorganisms that have increasingly been implicated to play key roles in health (Silpe & Balskus, 2021 ▸; Chang, 2020 ▸). Increasing evidence links dysbiosis in the microbiome to a variety of diseases/disorders, including inflammatory bowel disease and cancers (Mohseni *et al.*, 2020 ▸; Wilson *et al.*, 2019 ▸; Xue *et al.*, 2019 ▸; Bossuet-Greif *et al.*, 2018 ▸; Morgan *et al.*, 2022 ▸). A detailed understanding of microbe–microbe and host–microbe interactions would be useful towards general functional insights, along with approaches towards diagnosis, prevention and treatment. It has been established that certain strains of gut commensal bacteria produce a toxin, colibactin, that causes double-strand DNA breaks (Xue *et al.*, 2019 ▸; Dougherty & Jobin, 2021 ▸; Li *et al.*, 2019 ▸), promotes tumor formation in mouse models of colitis and is frequently found in patients with colorectal cancer (CRC; Dubinsky *et al.*, 2020 ▸). In addition to interactions between colibactin and mammalian cells, it has recently been demonstrated that colibactin targets the gut microbiome using a prophage-inducing mechanism leading to microbial cell lysis (Silpe *et al.*, 2022 ▸).

The colibactin biosynthetic pathway is encoded by a 54 kb gene cluster termed *clb* (or *pks*) centered around a hybrid non­ribosomal peptide synthetase–polyketide synthase (NRPS–PKS) machinery. Colibactin biosynthesis (Supplementary Fig. S1) involves a prodrug-like mechanism (Brother­ton & Balskus, 2013 ▸; Bian *et al.*, 2013 ▸; Volpe *et al.*, 2019 ▸) in which precolibactins are assembled in the cytoplasm and then transported to the periplasm by ClbM, a member of the MATE family of transporters (Mousa *et al.*, 2016 ▸, 2017 ▸). *N*-Deacylation of precolibactins by the periplasmic peptidase ClbP leads to the formation of the active genotoxin colibactin (Velilla *et al.*, 2023 ▸; Volpe *et al.*, 2023 ▸). Isolation and structure determination of colibactin has been a challenge because of product instability; however, several precolibactins have been isolated and the bioactive product has been characterized (Williams *et al.*, 2020 ▸; Tang *et al.*, 2022 ▸; Wernke *et al.*, 2020 ▸; Hirayama *et al.*, 2022 ▸). Complementary to this, *clb*+ *Escherichia coli* have been shown to generate DNA interstrand cross-links, both *in vivo* and *in vitro*, via N7 adenine alkylation by the electrophilic cyclopropane ring of colibactin (Wilson *et al.*, 2019 ▸; Xue *et al.*, 2019 ▸).

**Figure 1:**
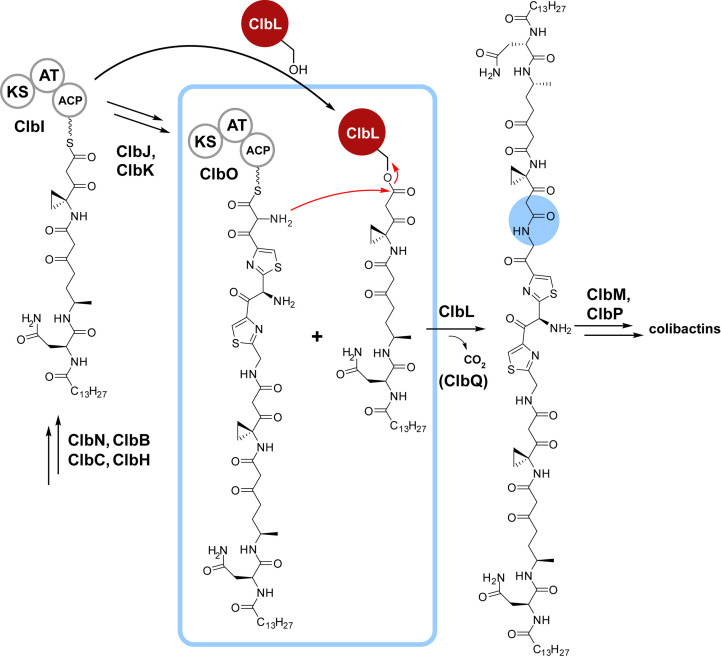
ClbL-mediated transacylation of colibactin NRPS–PKS intermediates. Phosphopantetheinyl thioesters of the ACP domains of ClbO and ClbI are substrates for ClbL transamidation. The bond-forming step (blue square) and product amide (blue circle) are highlighted.

*ClbL* is one of the five genes in the colibactin gene cluster that are found to be upregulated in CRC mouse models (Arthur *et al.*, 2012 ▸). Additionally, gene-deletion studies have implicated *clbL* as being required for the cytopathic effects of colibactin (Nougayrède *et al.*, 2006 ▸). It has also been demonstrated that ClbL acts as an amide bond-forming enzyme and it has been proposed to be involved in the final coupling step in precolibactin biosynthesis (Fig. 1 ▸; Jiang *et al.*, 2019 ▸). The unique enzymatic transformation involves the formation of an amide bond between α-aminoketone and β-ketothioester acyl carrier protein (ACP) thioester intermediates, as demonstrated both *in vivo* and *in vitro* (Jiang *et al.*, 2019 ▸). ClbL transacylation produces the pseudodimeric precolibactin that is further elaborated to the mature genotoxin. Another distinctive aspect of ClbL is that the two substrates are both ACP-linked phosphopantetheinyl thioesters of two distinct intermediates along the NRPS–PKS biosynthetic assembly line.

ClbL is a member of the diverse amidase superfamily (AS) of enzymes that are characterized by a highly conserved Ser–*cis*-Ser–Lys catalytic triad (Supplementary Fig. S2) that is key to amide hydrolysis (Shin *et al.*, 2002 ▸; Valiña *et al.*, 2004 ▸; Patricelli & Cravatt, 2000 ▸; Labahn *et al.*, 2002 ▸). The general catalytic mechanism proceeds through an acylenzyme intermediate followed by nucleophilic substitution (commonly water for AS enzymes). In addition to the triad, AS enzymes contain a conserved stretch of approximately 130 amino acids termed the AS sequence that contains a core catalytic motif surrounded by α-helices. Enzymes of this family are widely found in both prokaryotes and eukaryotes, and exhibit a wide variety of functions with diverse substrate specificity; they include peptide amidase (Neumann *et al.*, 2002 ▸), fatty-acid amide hydrolase (Bracey *et al.*, 2002 ▸; Cravatt *et al.*, 1996 ▸), malonamidase E2 (Shin *et al.*, 2002 ▸) and glutamyl-tRNA amidotransferase subunit A (Nakamura *et al.*, 2006 ▸; Curnow *et al.*, 1997 ▸).

ClbL differs from the canonical AS family chemistry by linking an α-aminoketone to a β-ketothioester, resulting in the formation of an amide bond. The α-aminoketone nucleophile is conjugated to the carrier domain of ClbI and the β-ketothioester to that of ClbO. This specific acyl-transfer chemistry was supported by assaying various thioester- and amine-based substrates (Jiang *et al.*, 2019 ▸). The heterodimeric product from ClbL is subsequently hydrolyzed by ClbP, generating the bis-electrophile active product (Brotherton & Balskus, 2013 ▸; Bian *et al.*, 2013 ▸; Volpe *et al.*, 2019 ▸).

## 2. Methods

### 2.1. Cloning, expression and purification of ClbL

The *clbL* gene was cloned from a bacterial artificial chromosome harboring the colibactin *pks* island (Nougayrède *et al.*, 2006 ▸) into pET-28a (NdeI/XhoI sites; Supplementary Table S1). *clbL*-pET-28a was transformed into *E. coli* C43(DE3) and grown in LB–kanamycin medium at 37°C to an OD_600_ of ∼0.3 and then at 25°C to a OD_600_ of ∼0.6. 100 µ*M* isopropyl β-d-1-thiogalactopyranoside was added and growth was continued at 25°C for 16 h. The cells were harvested by centrifugation, resuspended in 20 m*M* Tris–HCl pH 8.0, 500 m*M* NaCl, 1 µg ml^−1^ aprotinin, 1 µg ml^−1^ pepstatin, 1 m*M* phenylmethylsulfonyl fluoride (PMSF) and lysed using a microfluidizer. Insoluble material was removed by centrifugation at 11 000 rev min^−1^ and the supernatant was incubated with 0.5 ml Ni–NTA resin for 1 h at 4°C. The resin was washed with 5× 10 ml 20 m*M* Tris–HCl pH 8.0, 500 m*M* NaCl, 20 m*M* imidazole and was then eluted with 3× 1.5 ml 20 m*M* Tris–HCl pH 8.0, 500 m*M* NaCl, 250 m*M* imidazole. The protein was dialyzed against 20 m*M* Tris–HCl pH 8.0, 100 m*M* NaCl, 1 m*M* β-mercaptoethanol, 10% glycerol and was purified using ion-exchange (HiTrap Q, GE Biosciences) and size-exclusion (HiLoad Superdex 75, GE Biosciences) chromatography.

### 2.2. Crystallization

ClbL was concentrated to 4.5 mg ml^−1^ and screened for crystallization in 96-well sitting-drop plates using commercial sparse-matrix screens. The initial crystallization conditions were optimized to 0.1 *M* Tris–HCl pH 8.5, 30% PEG 3000. The crystals were harvested and flash-cooled in liquid nitrogen, and data were collected on the 23-ID-D beamline at the Advanced Photon Source, Argonne National Laboratory. The 1.9 Å resolution diffraction data were indexed and scaled using the *XDS* package (Kabsch, 2010 ▸) and the structure was solved by molecular replacement using PDB entry 5h6s (27% sequence identity, 93% coverage; Akiyama *et al.*, 2017 ▸). The structure was refined to an *R*_work_ of 0.20 and an *R*_free_ of 0.25; overall refinement statistics are shown in Table 1 ▸. Manual and automated model building were iteratively performed using *Coot* (Emsley *et al.*, 2010 ▸) and real-space refinement in *Phenix* (Liebschner *et al.*, 2019 ▸). The *PyMOL* molecular-graphics system (version 2.0; Schrödinger) was used to generate graphical representations.

### 2.3. Molecular modeling

Protein docking was performed using the *LZerD* protein-docking webserver (https://lzerd.kiharalab.org/about/). *AlphaFold*-generated models of ClbL and the ACP domain of ClbO (residues 742–819) were used as input, with a default clustering cutoff of 4 Å (Venkatraman *et al.*, 2009 ▸; Senior *et al.*, 2020 ▸). The per-residue confidence scores (pLDDTs) for both *AlphaFold* models were >90 for the majority of the residues, except for a 38-residue stretch (324–362) in ClbL with a confidence score of 50–70. The top ten outputs from the *LZerD* server were all clustered above the ClbL active site with ranksum scores ranging from 87 to 407. The second-best docked model with a ranksum score of 110 was used for further analysis in Supplementary Fig. S6.

## 3. Results

### 3.1. Overall structure of ClbL

**Figure 2:**
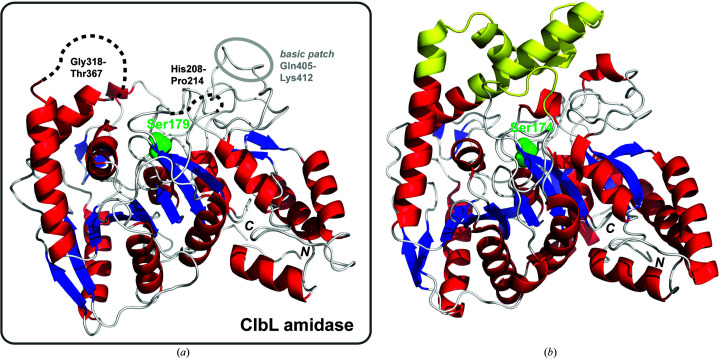
Structure of ClbL along with comparison with a representative member of the AS superfamily. (*a*) Overall protein structure highlighting Ser179 in the active site (green) along with two disordered loop regions and the basic loop. (*b*) Structure of a bacterial aryl acylamidase (Lee *et al.*, 2015 ▸) shown in the same orientation with the corresponding Ser174 highlighted.

To provide insight into its unique catalytic properties and substrate specificity, we determined the structure of ClbL at 1.9 Å resolution (Fig. 2 ▸ *a*). Based on comparative sequence analysis, a hydrazidase from *Microbacterium* (PDB entry 5h6s, 27% sequence identity; Schmitt *et al.*, 2005 ▸) was used as a molecular-replacement model to determine the structure. ClbL crystallized as a homodimer (space group *C*121) and a single polypeptide chain consists of 487 residues. Interpretable electron density for residues 318–367 and 208–214 is missing, suggesting flexible loop regions adjacent to and covering the active site. The overall structure displays a compact mixed α/β fold consisting of 12 α-helices and 12 β-strands. This general fold is similar to that observed in other members of the AS enzyme superfamily, and a structural homology-based search showed the highest similarities to glutamyl-tRNA amidotransferase (Schmitt *et al.*, 2005 ▸), malonamidase E2 (Shin *et al.*, 2002 ▸) and fatty-acid amide hydrolase (Cravatt *et al.*, 1996 ▸).

Compared with other members of the AS superfamily, a notable structural feature of ClbL is the presence of several disordered regions around the active site. A lack of electron density is evident in both monomers of the asymmetric unit, suggesting that the disordered loops are relevant to the protein in solution. AS family members, which process relatively small-molecule substrates, commonly have an ordered active-site region; bacterial aryl acylamidase (PDB entry 4yj6, 28% sequence identity; Lee *et al.*, 2015 ▸) exemplifies this difference (Fig. 2 ▸, Supplementary Fig. S3).

To explore the disordered active-site loop and ClbL–carrier domain interactions, we examined a model structure using *AlphaFold* (Senior *et al.*, 2020 ▸). The model is very similar to our experimental structure (Supplementary Fig. S4), with an r.m.s.d. on all atoms of 1.3 Å, and includes a model for the disordered regions adjacent to the active site that are not present in our experimental model. Residues 318–367 form a four-helix bundle that does not fully cover access to the active site. An extended helical structure could help to prevent hydrolysis chemistry and could also be involved in carrier-domain interactions. The arrangement of α-helices in the *AlphaFold* model is unlike that observed in canonical AS members such as aryl acylamidase (Fig. 2 ▸).

### 3.2. Active-site structure and substrate interactions

**Figure 3:**
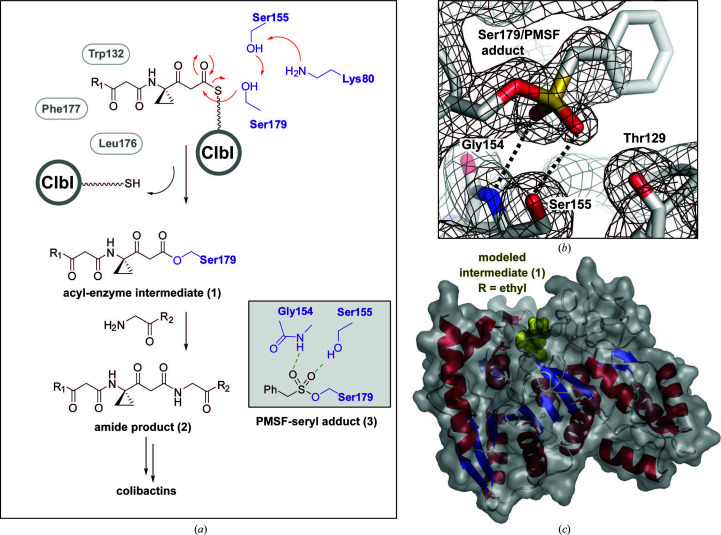
Substrate–ClbL active-site interactions. (*a*) Schematic of the overall ClbL reaction and substrate interactions. The overall pathway is shown along with the interactions of a modeled PMSF adduct (boxed). R_1_ and R_2_ represent the extended end chains of precolibactin (Fig. 1 ▸). (*b*) Composite omit electron-density map, contoured at 1.0 Å, of the modeled phenylmethylsulfonyl–Ser179 adduct. Hydrogen-bonding distances are 2.7 Å (Ser155/sulfonyl) and 3.2 Å (Ser155 *cis*-amide/sulfonyl). (*c*) ClbL surface representation of a modeled bound substrate (Supplementary Fig. S5, yellow).

The active-site catalytic triad of ClbL consists of Ser179–*cis*-Ser155–Lys80 (Fig. 3 ▸ *a*). The protein crystallized in an active conformation, as is evident from the covalent conjugation of an unanticipated small molecule to Ser179 in the active site. PMSF, which is present in the ClbL purification steps, models well into the orphan electron density (Fig. 3 ▸ *b*). Electron density corresponding to the phenyl group is not clear, suggesting disorder/nonspecific interactions. Both O atoms of the sulfonate group make hydrogen-bonding interactions with the side-chain hydroxyl of Ser155 and the *cis*-amide N atom of Ser155. These interactions could mimic an oxyanion hole-type stabilization of the tetrahedral intermediate of the reaction. Based on the conjugate-bound Ser179, the substrate β-ketothioester (Fig. 1 ▸, boxed ClbL-bound intermediate) was modeled into the active site (Fig. 3 ▸ *c*, Supplementary Fig. S5). An extended conjugate, as modeled, is in position to form a hydrogen bond between the β carbonyl group and the backbone amide of Phe177. Additionally, Trp132 and Leu176 are in position to form a hydrophobic pocket to accommodate the aliphatic portion of the substate. The overall conformation places the substrate extending to the surface of the enzyme.

The substrates of ClbL are unique compared with other AS superfamily enzymes. The observed substrate-binding pocket is largely hydrophobic and is of a suitable size to accommodate the two predicted substrates. The hydrophobicity of the binding pocket along with an ∼50-residue flexible lid can disfavor water from the catalytic site while favoring conjugation over amidase chemistry. Based on the previous reports and our structural data, we hypothesize that Lys80 acts as a general base and abstracts a proton from Ser155, which in turn activates Ser179 for nucleophilic attack on the β-ketothioester intermediate (Jiang *et al.*, 2019 ▸). The resulting tetrahedral intermediate forms an acyl-enzyme complex stabilized by hydrogen-bonding interactions with the backbone amides of Gly154 and Ser155 (Fig. 3 ▸ *b*, Supplementary Fig. S5). The general chemistry of the AS superfamily involves water reacting with an acyl-enzyme intermediate to produce a hydrolysis product. In the absence of water, a properly oriented nucleophile can readily react with the acyl-enzyme complex (Goswami & Van Lanen, 2015 ▸). In contrast, ClbL catalyzes α-aminoketone addition to a serine-bound acyl-enzyme intermediate, resulting in the formation of an amide bond (Fig. 3 ▸ *a*), an exothermic reaction.

### 3.3. Predicted interactions with partner carrier domains

ACP domains are small proteins consisting of four α-helices, generally with a low isoelectric point (pI). ACP–partner enzyme interactions are commonly based on electrostatic interactions (Moretto *et al.*, 2017 ▸; Keatinge-Clay, 2016 ▸). The specificity of *in trans* interactions of ClbL with the ACP domains of ClbI and ClbO was examined using sequence analysis. From our structure, the disordered/partially disordered loop regions of ClbL are predominately basic, with an overall negative charge. This is exemplified by a loop (residues 405–412; N-QQPVRKRK) and a unmodeled loop (318–367) with an estimated pI of 9.5. The ACP domain commonly interacts with partner enzymes through interactions of helix 2 and the preceding loop region, and there is not a common interaction mode among representative examples (Gulick & Aldrich, 2018 ▸). The post-translationally modified serine residue is located at the N-terminus of helix 2. The overall charge of both ClbI and ClbO is negative, with pIs of ∼4.0 and ∼4.9, respectively. ClbO has prominent negatively charged patches, for example EHSEFISECVD; this general spacing of side chains suggests that the acid groups are on the same face of helix 2.

The chemistry of ClbL on the carrier domain of ClbO is non­canonical (amide-bond formation) when compared with PKS carrier-domain enzyme transformations. From looking at sequence differences between the eight carrier domains in the colibactin assembly line, the carrier domain of ClbO more closely resembles peptidyl carrier proteins despite being in a PKS module. To provide supporting evidence for ClbL–carrier domain interactions, we modeled carrier-domain interactions with ClbL using the described *AlphaFold* model (a second-order analysis) to dock the carrier domain of ClbO (Supplementary Figs. S6 and S7). The ACP was placed in a productive orientation to deliver a phosphopantetheinyl substrate into the active site. The distance between the modified ClbO serine residue and the active site of ClbL is 22 Å, which is within the distance for established carrier domain–enzyme interactions. In addition, the electrostatic interactions predicted by sequence alignment are supported by the modeled didomain structure, with residues 405–412 (QQPVRKRK) in close proximity to helix 2 of ClbO (EHSEFISECVD) and with acidic residues on one face of the helix close to the basic loop of ClbL.

Amidases are ubiquitous enzymes that exhibit a wide variety of functions, including the hydrolysis of a wide range of amide substrates including short-chain aliphatic amides, mid-chain amides, arylamides, α-aminoamides and α-hydroxy­amides. To understand the unusual transacylation activity of ClbL, we created a sequence-similarity network (SSN; Gerlt *et al.*, 2015 ▸) for family PF01425 (Supplementary Fig. S8). The SSN diagram shows clustering of ClbL from different *pks*+ species in a clade distinct from other representative amidases, further supporting a unique catalytic role of ClbL.

## 4. Conclusions

Overall, this work provides a structural basis for the unique chemistry of ClbL, which is a key biosynthetic step in the formation of the heterodimeric precolibactin with two cyclopropane warheads. Based on the structure of an active-site adduct, the substrate was modeled into the active site. Additionally, model carrier protein–ClbL interactions were proposed, supporting the role of ClbL in the biosynthetic assembly-line pathway to colibactin.

## Supplementary Material

PDB reference: ClbL, 8es6

Supplementary Figures and Tables. DOI: 10.1107/S2059798323005703/ag5043sup1.pdf

## Figures and Tables

**Table 1:** Structure-refinement statistics for ClbL (PDB entry 8es6) Values in parentheses are for the highest resolution shell.

Wavelength (Å)	1.033
Temperature (K)	100
Resolution range (Å)	49.55–1.90 (1.968–1.900)
Space group	*C*121
*a*, *b*, *c* (Å)	103.9, 56.4, 145.0
α, β, γ (°)	90, 91.8, 90
Total reflections	439975 (42943)
Unique reflections	66423 (6614)
Multiplicity	6.6 (6.5)
Completeness (%)	99.88 (99.92)
Mean *I*/σ(*I*)	10.74 (1.49)
Wilson *B* factor (Å^2^)	24.98
*R*_merge_	0.1258 (1.162)
*R*_meas_	0.137
CC_1/2 _	0.996 (0.730)
CC*	0.999 (0.919)
*R*_work_	0.2063 (0.3169)
*R*_free_	0.2489 (0.3548)
No. of non-H atoms	
Total	6969
Macromolecules	6711
Ligands	0
Water	258
Protein residues	858
R.m.s.d., bond angles (Å)	0.008
R.m.s.d., angles (°)	1.01
Ramachandran favored (%)	95.97
Ramachandran outliers (%)	0.0
Clashscore	6.0
Average *B* factor (Å^2^)	
Overall	34.58
Macromolecules	34.71
Solvent	31.04

## References

[bb2] Akiyama, T., Ishii, M., Takuwa, A., Oinuma, K. I., Sasaki, Y., Takaya, N. & Yajima, S. (2017). *Biochem. Biophys. Res. Commun.* **482**, 1007–1012. 10.1016/j.bbrc.2016.11.148 27908731

[bb3] Arthur, J. C., Perez-Chanona, E., Mühlbauer, M., Tomkovich, S., Uronis, J. M., Fan, T. J., Campbell, B. J., Abujamel, T., Dogan, B., Rogers, A. B., Rhodes, J. M., Stintzi, A., Simpson, K. W., Hansen, J. J., Keku, T. O., Fodor, A. A. & Jobin, C. (2012). *Science*, **338**, 120–123. 10.1126/science.1224820 PMC3645302 22903521

[bb4] Bian, X., Fu, J., Plaza, A., Herrmann, J., Pistorius, D., Stewart, A. F., Zhang, Y. & Müller, R. (2013). *ChemBioChem*, **14**, 1194–1197. 10.1002/cbic.201300208 23744512

[bb5] Bossuet-Greif, N., Vignard, J., Taieb, F., Mirey, G., Dubois, D., Petit, C., Oswald, E. & Nougayrède, J.-P. (2018). *mBio*, **9**, e02393-17. 10.1128/mBio.02393-17 PMC5874909 29559578

[bb6] Bracey, M. H., Hanson, M. A., Masuda, K. R., Stevens, R. C. & Cravatt, B. F. (2002). *Science*, **298**, 1793–1796. 10.1126/science.1076535 12459591

[bb7] Brotherton, C. A. & Balskus, E. P. (2013). *J. Am. Chem. Soc.* **135**, 3359–3362. 10.1021/ja312154m 23406518

[bb8] Chang, P. V. (2020). *ACS Chem. Biol.* **15**, 1119–1126. 10.1021/acschembio.9b00813 PMC8218590 31895538

[bb9] Cravatt, B. F., Giang, D. K., Mayfield, S. P., Boger, D. L., Lerner, R. A. & Gilula, N. B. (1996). *Nature*, **384**, 83–87. 10.1038/384083a0 8900284

[bb10] Curnow, A. W., Hong, K., Yuan, R., Kim, S., Martins, O., Winkler, W., Henkin, T. M. & Söll, D. (1997). *Proc. Natl Acad. Sci. USA*, **94**, 11819–11826. 10.1073/pnas.94.22.11819 PMC23611 9342321

[bb11] Dougherty, M. W. & Jobin, C. (2021). *Toxins*, **13**, 346. 10.3390/toxins13050346 PMC8151066 34065799

[bb12] Dubinsky, V., Dotan, I. & Gophna, U. (2020). *Trends Microbiol.* **28**, 874–876. 10.1016/j.tim.2020.05.015 32507544

[bb13] Emsley, P., Lohkamp, B., Scott, W. G. & Cowtan, K. (2010). *Acta Cryst.* D**66**, 486–501. 10.1107/S0907444910007493 PMC2852313 20383002

[bb14] Gerlt, J. A., Bouvier, J. T., Davidson, D. B., Imker, H. J., Sadkhin, B., Slater, D. R. & Whalen, K. L. (2015). *Biochim. Biophys. Acta*, **1854**, 1019–1037. 10.1016/j.bbapap.2015.04.015 PMC4457552 25900361

[bb15] Goswami, A. & Van Lanen, S. G. (2015). *Mol. Biosyst.* **11**, 338–353. 10.1039/c4mb00627e PMC4304603 25418915

[bb16] Gulick, A. M. & Aldrich, C. C. (2018). *Nat. Prod. Rep.* **35**, 1156–1184. 10.1039/c8np00044a PMC6235721 30046790

[bb17] Hirayama, Y., Sato, M. & Watanabe, K. (2022). *Biochemistry*, **61**, 2782–2790. 10.1021/acs.biochem.2c00229 35723977

[bb18] Jiang, Y., Stornetta, A., Villalta, P. W., Wilson, M. R., Boudreau, P. D., Zha, L., Balbo, S. & Balskus, E. P. (2019). *J. Am. Chem. Soc.* **141**, 11489–11496. 10.1021/jacs.9b02453 PMC6728428 31251062

[bb19] Kabsch, W. (2010). *Acta Cryst.* D**66**, 125–132. 10.1107/S0907444909047337 PMC2815665 20124692

[bb20] Keatinge-Clay, A. T. (2016). *Nat. Prod. Rep.* **33**, 141–149. 10.1039/c5np00092k PMC4742408 26584443

[bb21] Labahn, J., Neumann, S., Büldt, G., Kula, M. R. & Granzin, J. (2002). *J. Mol. Biol.* **322**, 1053–1064. 10.1016/s0022-2836(02)00886-0 12367528

[bb22] Lee, S., Park, E.-H., Ko, H.-J., Bang, W. G., Kim, H.-Y., Kim, K. H. & Choi, I.-G. (2015). *Biochem. Biophys. Res. Commun.* **467**, 268–274. 10.1016/j.bbrc.2015.09.177 26454172

[bb23] Li, Z.-R., Li, J., Cai, W., Lai, J. Y. H., McKinnie, S. M. K., Zhang, W.-P., Moore, B. S., Zhang, W. & Qian, P.-Y. (2019). *Nat. Chem.* **11**, 880–889. 10.1038/s41557-019-0317-7 PMC6761029 31527851

[bb1] Liebschner, D., Afonine, P. V., Baker, M. L., Bunkóczi, G., Chen, V. B., Croll, T. I., Hintze, B., Hung, L.-W., Jain, S., McCoy, A. J., Moriarty, N. W., Oeffner, R. D., Poon, B. K., Prisant, M. G., Read, R. J., Richardson, J. S., Richardson, D. C., Sammito, M. D., Sobolev, O. V., Stockwell, D. H., Terwilliger, T. C., Urzhumtsev, A. G., Videau, L. L., Williams, C. J. & Adams, P. D. (2019). *Acta Cryst.* D**75**, 861–877.

[bb24] Mohseni, A. H. S., Taghinezhad-S, S. & Fu, X. (2020). *Microb. Pathog.* **149**, 104569. 10.1016/j.micpath.2020.104569 33075518

[bb25] Moretto, L., Vance, S., Heames, B. & Broadhurst, R. W. (2017). *Chem. Commun.* **53**, 11457–11460. 10.1039/c7cc04625a PMC6038798 28980673

[bb26] Morgan, R. N., Saleh, S. E., Farrag, H. A. & Aboulwafa, M. M. (2022). *Crit. Rev. Microbiol.* **48**, 42–66. 10.1080/1040841X.2021.1944052 34265231

[bb27] Mousa, J. J., Newsome, R. C., Yang, Y., Jobin, C. & Bruner, S. D. (2017). *Biochem. Biophys. Res. Commun.* **482**, 1233–1239. 10.1016/j.bbrc.2016.12.018 27939886

[bb28] Mousa, J. J., Yang, Y., Tomkovich, S., Shima, A., Newsome, R. C., Tripathi, P., Oswald, E., Bruner, S. D. & Jobin, C. (2016). *Nat. Microbiol.* **1**, 15009. 10.1038/nmicrobiol.2015.9 PMC5704960 27571755

[bb29] Nakamura, A., Yao, M., Chimnaronk, S., Sakai, N. & Tanaka, I. (2006). *Science*, **312**, 1954–1958. 10.1126/science.1127156 16809541

[bb30] Neumann, S., Granzin, J., Kula, M.-R. & Labahn, J. (2002). *Acta Cryst.* D**58**, 333–335. 10.1107/s0907444901020248 11807268

[bb31] Nougayrède, J.-P., Homburg, S., Taieb, F., Boury, M., Brzuszkiewicz, E., Gottschalk, G., Buchrieser, C., Hacker, J., Dobrindt, U. & Oswald, E. (2006). *Science*, **313**, 848–851. 10.1126/science.1127059 16902142

[bb32] Patricelli, M. P. & Cravatt, B. F. (2000). *J. Biol. Chem.* **275**, 19177–19184. 10.1074/jbc.M001607200 10764768

[bb33] Schmitt, E., Panvert, M., Blanquet, S. & Mechulam, Y. (2005). *Structure*, **13**, 1421–1433. 10.1016/j.str.2005.06.016 16216574

[bb34] Senior, A. W., Evans, R., Jumper, J., Kirkpatrick, J., Sifre, L., Green, T., Qin, C., Žídek, A., Nelson, A. W. R., Bridgland, A., Penedones, H., Petersen, S., Simonyan, K., Crossan, S., Kohli, P., Jones, D. T., Silver, D., Kavukcuoglu, K. & Hassabis, D. (2020). *Nature*, **577**, 706–710. 10.1038/s41586-019-1923-7 31942072

[bb35] Shin, S., Lee, T.-H., Ha, N.-C., Koo, H. M., Kim, S., Lee, H.-S., Kim, Y. S. & Oh, B.-H. (2002). *EMBO J.* **21**, 2509–2516. 10.1093/emboj/21.11.2509 PMC126024 12032064

[bb36] Silpe, J. E. & Balskus, E. P. (2021). *ACS Cent. Sci.*, **7**, 20–29. 10.1021/acscentsci.0c01030 PMC7844856 33532566

[bb37] Silpe, J. E., Wong, J. W. H., Owen, S. V., Baym, M. & Balskus, E. P. (2022). *Nature*, **603**, 315–320. 10.1038/s41586-022-04444-3 PMC8907063 35197633

[bb38] Tang, J.-W., Liu, X., Ye, W., Li, Z.-R. & Qian, P.-Y. (2022). *Nat. Prod. Rep.* **39**, 991–1014. 10.1039/d1np00050k 35288725

[bb39] Valiña, A. L., Mazumder-Shivakumar, D. & Bruice, T. C. (2004). *Biochemistry*, **43**, 15657–15672. 10.1021/bi049025r 15595822

[bb40] Velilla, J. A., Volpe, M. R., Kenney, G. E., Walsh, R. M. Jr, Balskus, E. P. & Gaudet, R. (2023). *Nat. Chem. Biol.* **19**, 151–158. 10.1038/s41589-022-01142-z PMC9889268 36253550

[bb41] Venkatraman, V., Yang, Y. D., Sael, L. & Kihara, D. (2009). *BMC Bioinformatics*, **10**, 407. 10.1186/1471-2105-10-407 PMC2800122 20003235

[bb42] Volpe, M. R., Velilla, J. A., Daniel-Ivad, M., Yao, J. J., Stornetta, A., Villalta, P. W., Huang, H.-C., Bachovchin, D. A., Balbo, S., Gaudet, R. & Balskus, E. P. (2023). *Nat. Chem. Biol.* **19**, 159–167. 10.1038/s41589-022-01147-8 PMC9889270 36253549

[bb43] Volpe, M. R., Wilson, M. R., Brotherton, C. A., Winter, E. S., Johnson, S. E. & Balskus, E. P. (2019). *ACS Chem. Biol.* **14**, 1097–1101. 10.1021/acschembio.9b00069 PMC6726110 31059217

[bb44] Wernke, K. M., Xue, M., Tirla, A., Kim, C. S., Crawford, J. M. & Herzon, S. B. (2020). *Bioorg. Med. Chem. Lett.* **30**, 127280. 10.1016/j.bmcl.2020.127280 PMC7309967 32527463

[bb45] Williams, P. C., Wernke, K. M., Tirla, A. & Herzon, S. B. (2020). *Nat. Prod. Rep.* **37**, 1532–1548. 10.1039/d0np00072h PMC7700718 33174565

[bb46] Wilson, M. R., Jiang, Y., Villalta, P. W., Stornetta, A., Boudreau, P. D., Carrá, A., Brennan, C. A., Chun, E., Ngo, L., Samson, L. D., Engelward, B. P., Garrett, W. S., Balbo, S. & Balskus, E. P. (2019). *Science*, **363**, eaar7785. 10.1126/science.aar7785 PMC6407708 30765538

[bb47] Xue, M., Kim, C. S., Healy, A. R., Wernke, K. M., Wang, Z., Frischling, M. C., Shine, E. E., Wang, W., Herzon, S. B. & Crawford, J. M. (2019). *Science*, **365**, eaax2685. 10.1126/science.aax2685 PMC6820679 31395743

