# Supplementary material for: Structural basis of the amidase ClbL central to the biosynthesis of the genotoxin colibactin
Source: Acta Crystallogr D Struct Biol. 2023 Aug 10;79(Pt 9):830–6. doi: 10.1107/S2059798323005703 (PMC10478638; doi:10.1107/S2059798323005703)
Supplement: Supplementary file 1 [file d-79-00830-sup1.pdf]

STRUCTURAL
BIOLOGY

Volume 79 (2023)

Supporting information for article:

Structural basis of the amidase ClbL central to the biosynthesis of the genotoxin colibactin

Prabhanshu Tripathi, Jarrod Mousa, Naga Sandhya Guntaka and Steven Bruner

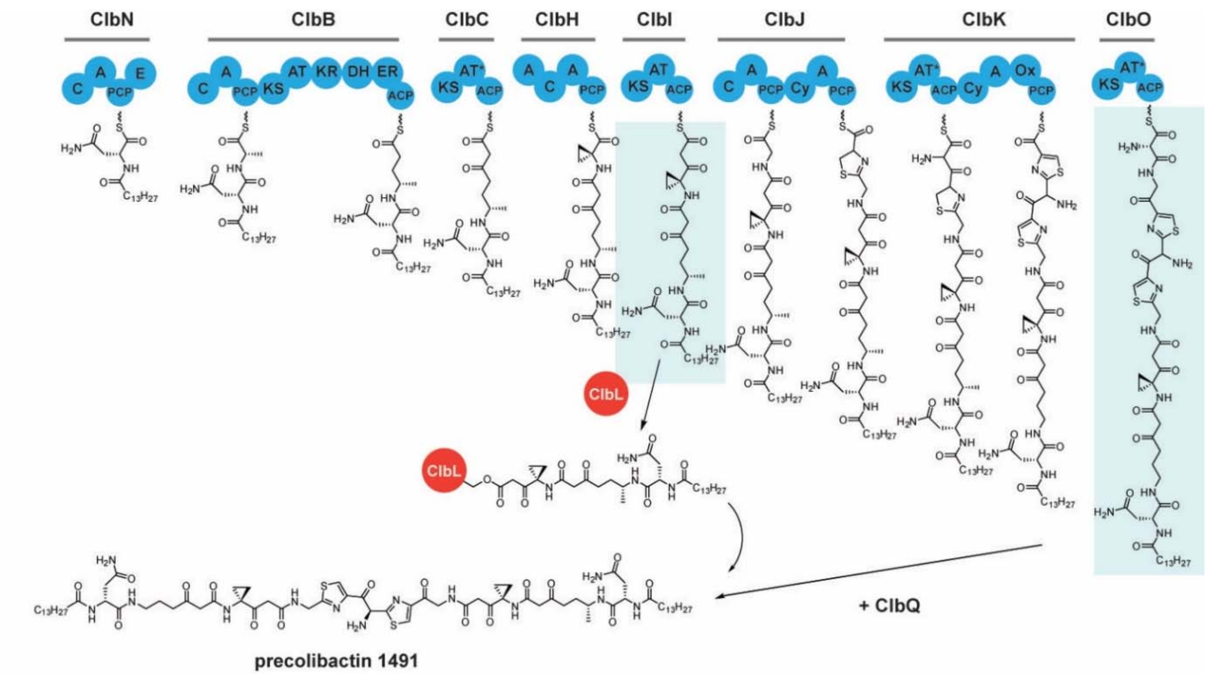

Figure S1 Proposed colibactin biosynthetic pathway. The NRPS/PKS assembly line is illustrated leading to precolibactin 1491. The role of ClbL is highlighted, with the substrate assembly line intermediates highlighted (light blue) along with the ClbL-covalent adduct intermediate. Precolibactin 1491 is further processed to generate to active compound (not shown).

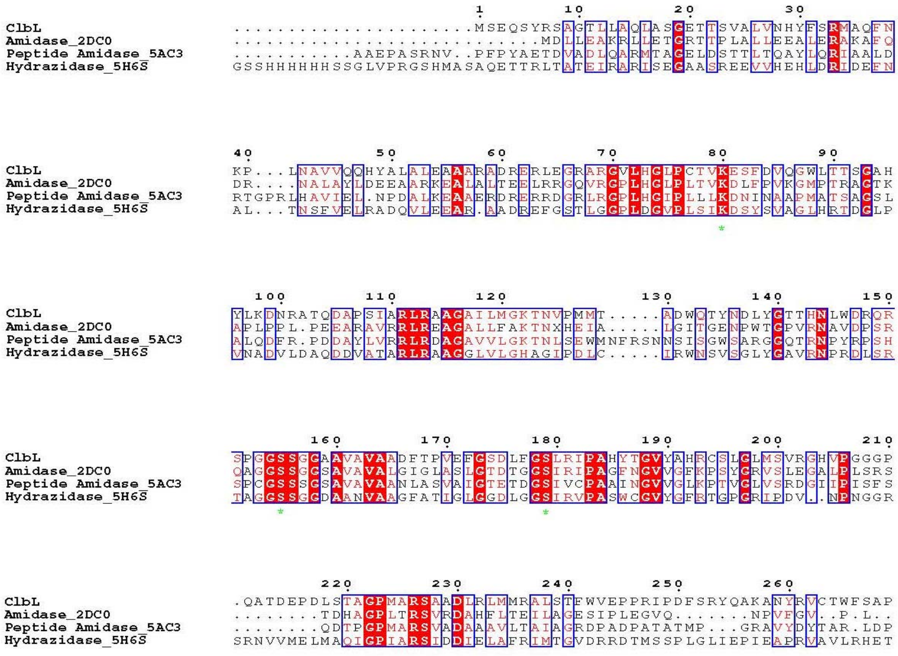

Figure S2 Sequence alignment of ClbL with select amidase superfamily members. The black arrows show the conserved Ser-cisSer-Lys catalytic triad.

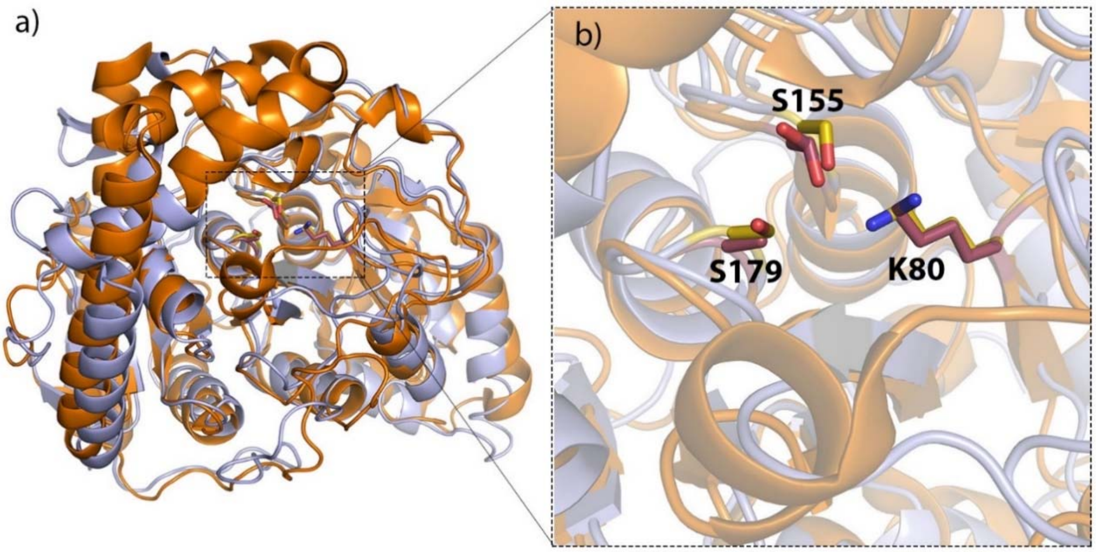

Figure S3 A. ClbL (light blue) structural alignment with the molecular replacement model hydrazidase (orange, PDB: 5H6S). B. Overlay of the active site with the catalytic triad shown.

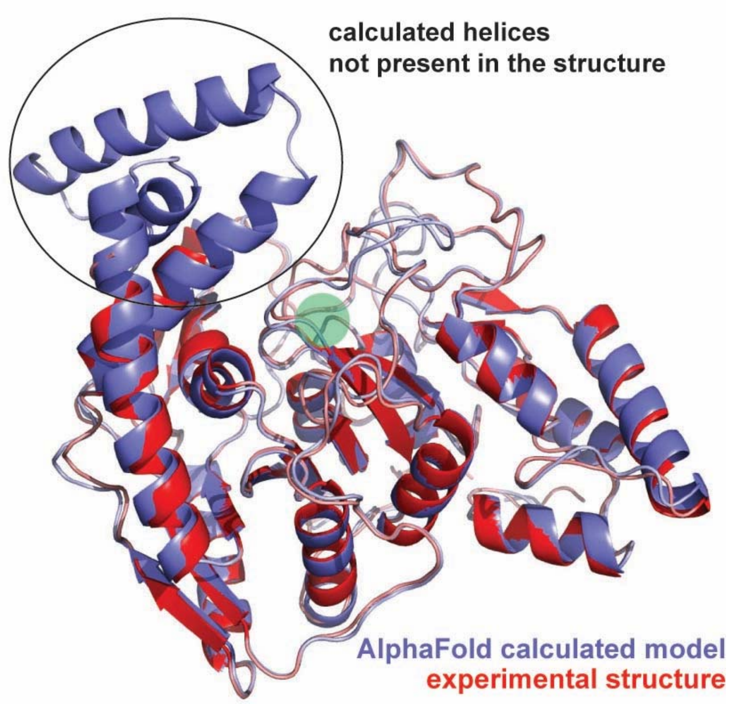

Figure S4 Comparison of the ClbL structure (red) with a calculated model (blue) using Alphafold. The active site is indicated (green).

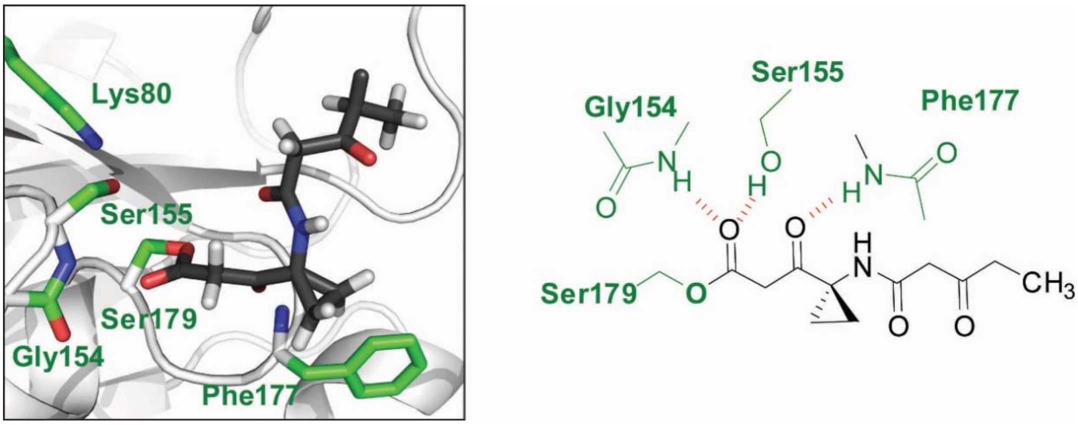

Figure S5 Substrate modelling in the active site based on the ClbL-PMSF adduct structure along with representation of the acyl-enzyme complex stabilized by hydrogen bonding interactions in the active site.

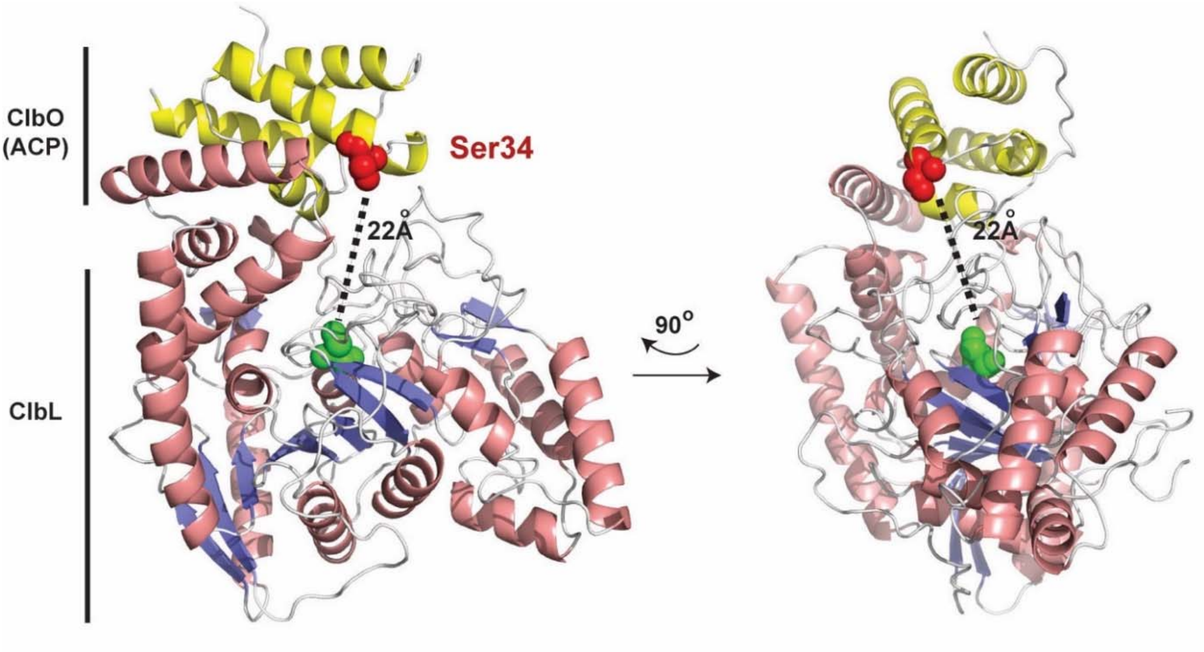

Figure S6 Modeling of ClbO/ClbL interactions. An Alphafold generated model of ClbO was docked onto ClbL using the LZerD web-based server. The docking places the post-translationally modified Ser34/ClbO at a distance of 22 Å from Ser179/ClbL.

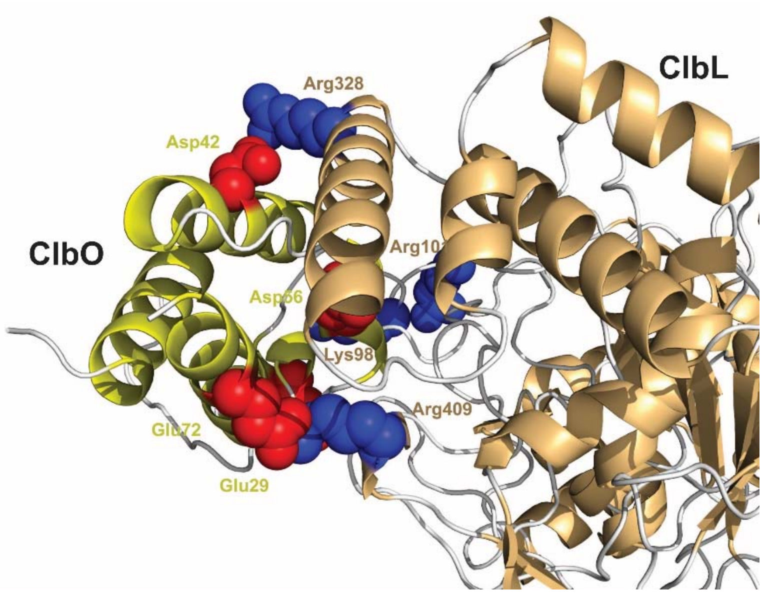

Figure S7 Interface of the ClbO/ClbL interaction based on protein modelling and docking results. Ionic/salt bridge interactions are highlighted as negatively charged (red, ClbO) and positively charged (blue, ClbL) residues.

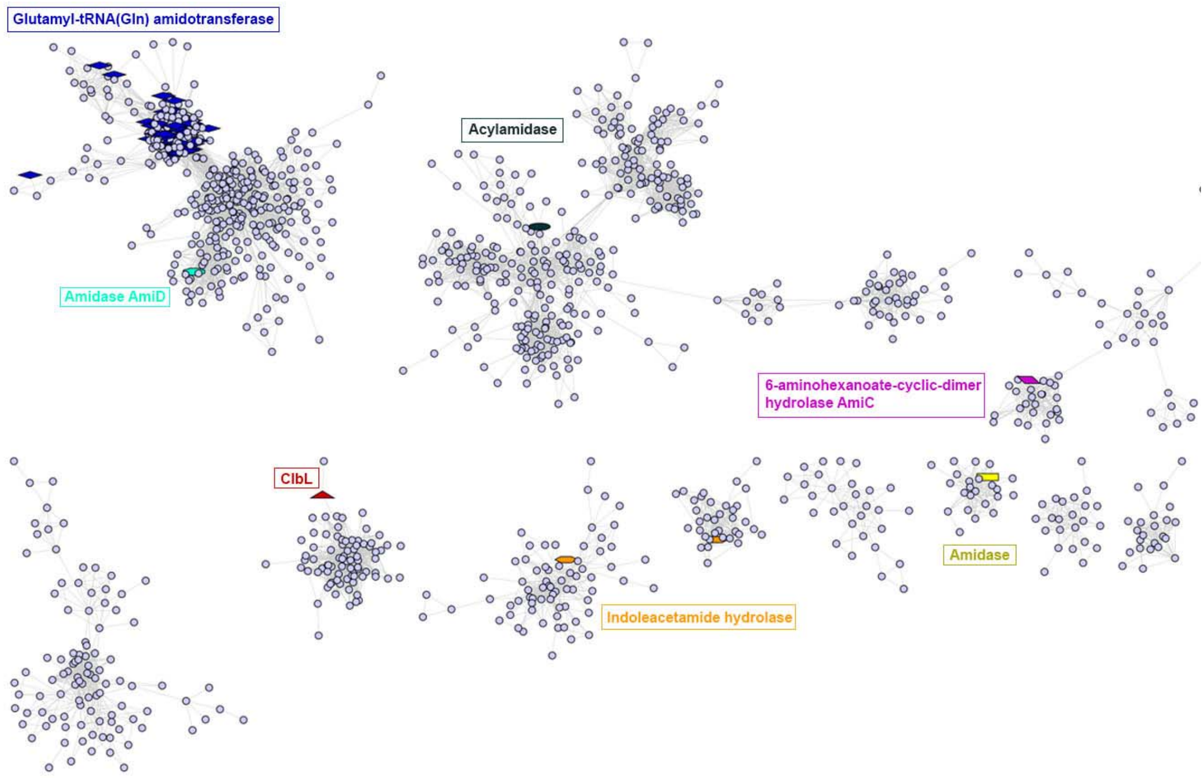

Figure S8 Sequence Similarity Network (SSN) diagram for PF01425. SSN created using UniRef50 with an alignment score (AS) of 86 shows ClbL from pks+ species lies in a distinct clade from representative amidases.

Table S1 List of DNA primers for cloning ClbL

#	Protein	Direction	Sequence
1.	pET28a-ClbL	Forward/ NdeI	CGGAGCCATATGAGTGAGCAGAGCTATCGT
2.	pET28a-ClbL	Reverse/ XhoI	GGCAGCCTCGAGCTAGTACCCTTCCGGTACCGTGAA
3.	pET30a-ClbL	Forward/ NdeI	CGGAGCCATATGAGTGAGCAGAGCTATCGT
4.	pET30a-ClbL	Reverse/ XhoI	GGCAGCCTCGAGGTACCCTTCCGGTACCGTGAA
